# The CT target sign as a criterion for the differential diagnosis between tuberculosis and organizing pneumonia

**DOI:** 10.1590/0037-8682-0095-2025

**Published:** 2025-07-07

**Authors:** Elcio Bakowski, Gláucia Zanetti, Edson Marchiori

**Affiliations:** 1 Hospital Vivalle, São José dos Campos, SP, Brasil.; 2 Universidade Federal do Rio de Janeiro, Rio de Janeiro, RJ, Brasil.

**FIGURE 1:**
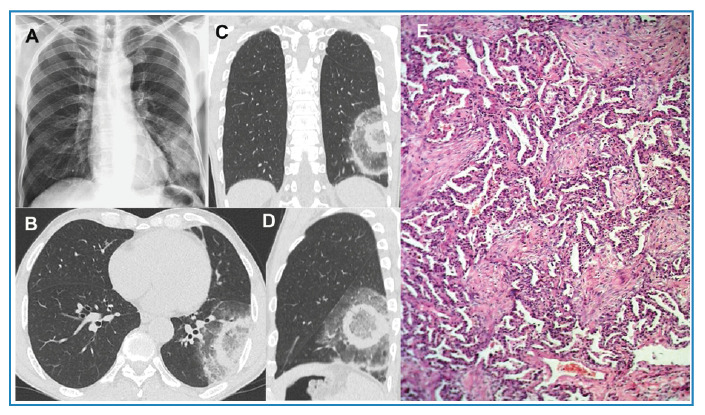
**(A)** Chest radiograph showing inhomogeneous opacities in the left lower lobe of the lung. Non-enhanced chest CT images with axial **(B)**, coronal **(C)** and sagittal **(D)** reconstruction demonstrate a focal core ground-glass opacity and two ring-like opacities immediately surrounding it, with the appearance of a shooting target (the CT target sign). A photomicrograph of the lung biopsy **(E)** shows the involvement of airspaces by polypoid fibroblastic foci distributed within terminal bronchioles, alveolar ducts, and alveoli (hematoxylin and eosin stain, ×40).

**FIGURE 2:**
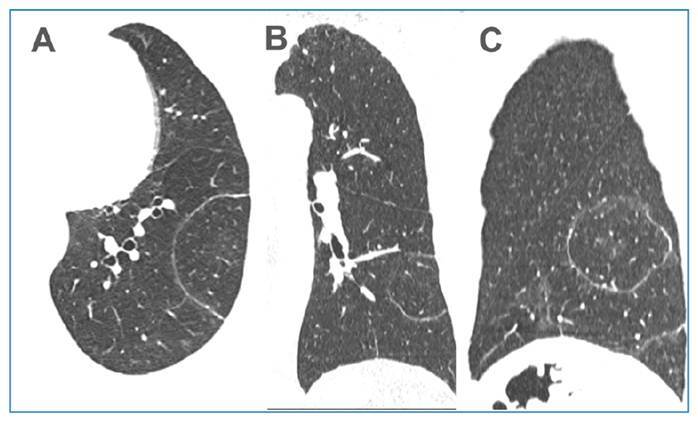
Non-enhanced chest CT images acquired three weeks after the first examination with axial **(A)**, coronal **(B)** and sagittal **(C)** reconstruction show marked resorption of the opacities, with only a dense halo persisting, involving the normal lung parenchyma.

A 61-year-old male patient developed fever, dry cough, myalgia, headache, and arthralgia and was treated with prednisone and symptom-based medications. The decision to initiate corticosteroid therapy instead of antibiotics was based on the atypical clinical and laboratory features of a bacterial infection, which led us to consider it an inflammatory or autoimmune disease. Five days later, the patient’s condition worsened and was hospitalized. The patient denied any other signs or symptoms. Complementary examinations including a respiratory viral panel yielded no significant findings. Chest radiography revealed inhomogeneous opacities in the left lower lobe of the lung (Figure 1A ). Chest CT findings were compatible with the target sign, which was interpreted as organizing pneumonia (Figure 1B-D). The patient underwent bronchoscopy, with bronchoalveolar lavage and biopsy. Real-time PCR (GeneXpert) was performed on bronchoalveolar lavage samples, and *Mycobacterium tuberculosis* was detected. A regimen consisting of RIPE (rifampin, isoniazid, pyrazinamide, and ethambutol) was initiated. Five days later, transbronchial biopsy showed a histological pattern of organizing pneumonia (Figure 1E), confirming the tomographic diagnosis. The RIPE regimen was considered a false-positive result and suspended based on the tomographic findings of the target sign and the results of the transbronchial biopsy; then, prednisone was started. The patient was discharged from hospital in good clinical condition. Three weeks after the initial examination, a control CT scan showed a marked reduction in the lesions (Figure 2).

The target sign consists of a central nodular opacity with variable densities (soft tissue or ground glass) surrounded by a less dense rim of either normal parenchyma or ground glass opacity. This is further encircled by a denser peripheral rim of ground glass or soft tissue-type density. In some cases, multiple concentric ring-like opacities may be observed^1^ ^-^ ^3^. In this instance, recognizing the target sign strongly suggests a diagnosis of organizing pneumonia^3^ ^-^ ^5^. To our knowledge, this sign has not been documented in patients with pulmonary tuberculosis. 
